# Ashwagandha (*Withania somnifera*) targets liver cancer stem cells via inhibiting Hedgehog signaling pathway in hepatocellular carcinoma

**DOI:** 10.1007/s12032-025-03215-0

**Published:** 2026-02-05

**Authors:** Samah E. Ismail, Amany I. Youssef, Esraa E. Fawzy, Eman A. Khalifa, Amani H. Kazem, Marwa A. Kholief, Zainab A. Saleh

**Affiliations:** 1 Applied Medical Chemistry Department, Medical Research Institute, Alexandria University, Alexandria, Egypt; 2 Botany and Microbiology Department, Faculty of Science, Alexandria University, Alexandria, Egypt; 3 Histochemistry and Cell Biology Department, Medical Research Institute, Alexandria University, Alexandria, Egypt; 4 Pathology Department, Medical Research Institute, Alexandria University, Alexandria, Egypt; 5 Forensic Medicine and Clinical Toxicology Department, Faculty of Medicine, Alexandria University, Alexandria, Egypt; 6 Center of Excellence for Research in Regenerative Medicine and Applications (CERRMA), Faculty of Medicine, Alexandria University, Alexandria, Egypt

**Keywords:** Ashwagandha, Sorafenib, Liver cancer stem cells, Hedgehog signaling pathway

## Abstract

Ashwagandha (*W. somnifera*), known for its broad health benefits, has shown potential in cancer prevention and treatment. The aim of this study was to investigate the potential effect of ashwagandha aqueous extract (ASH-AE) on cell proliferation and therapy resistance markers in hepatocellular carcinoma (HCC). An in vitro study was conducted using the HepG2 cell line. The HepG2 cells were divided into 4 groups according to the treatment regimen received. ASH-AE was extracted from the whole plant and characterized by GC-MS and HPLC. HepG2 cell viability was determined for all groups. The protein expression of cluster of differentiation 90 (CD90) was determined by flow cytometry technique. Also, the gene expression of Sonic Hedgehog (SHH), Patched 1(PTCH1) and ATP-binding cassette subfamily C1 (ABCC1) was assayed by qRT-PCR. The protein expression and localization of glioma-associated oncogene 1 (Gli1) in HepG2 cells were determined by immunocytochemistry (ICC) assay. The results indicated that ASH-AE either alone or in combination with sorafenib (SOR) significantly reduced HepG2 cell viability in a concentration dependent manner (P ˂0.001) with IC50 was 6.65 mg/ml for ASH-AE, 11.3 µM for SOR, and 5.6 mg/ml + 18.6 µM for ASH-AE in combination with SOR. Moreover, SOR significantly increased the percentage of CD90^+^ cells and Gli1 protein expression and nuclear translocation as well as ABCC1 gene expression compared to untreated cells. On the other hand, ASH-AE either alone or in combination with SOR significantly decreased the percentage of CD90^+^ cells and Gli1 expression and nuclear translocation as well as SHH, PTCH1 and ABCC1 gene expression compared to untreated cells and that treated with SOR. We concluded for the first time that the combination of SOR and ASH-AE generates antagonistic antitumor effect in HepG2 cells. Moreover, ASH-AE can inhibit proliferation of HepG2 cells and mitigate sorafenib-induced resistance-associated markers in HepG2 cells by targeting CD90^+^ cells via Hedgehog pathway modulation.

## Background

Hepatocellular carcinoma represents the most prevalent form of primary liver cancer. In terms of prevalence, HCC ranks as the sixth most common cancer globally (1). The prognosis for patients with advanced-stage HCC is generally poor due to the limited treatment options available (2). Systemic treatments, particularly those involving traditional cytotoxic agents, are often ineffective. Sorafenib has been the only systemic therapy shown to have clinical efficacy in managing advanced HCC for over a decade (3). However, the survival benefit from SOR is modest, extending life by only about three months, and only one-third of patients with advanced HCC respond to this treatment due to the development of SOR resistance (4).

Previously, it was indicated that cancer stem cells (CSCs) contribute to therapeutic resistance in HCC (5). CSC markers can predict responses to Sorafenib; for example, higher expression levels of CD90 and CD133 in HCC are associated with a poorer response to Sorafenib compared to normal expression levels of these markers (5).

Stemness-related signaling pathways such as Notch, Hippo, Wnt, and Hedgehog (Hh) are often aberrantly activated in CSCs, and blocking these pathways can overcome drug resistance (6). Activation of Hh pathway has been linked to both the initiation and early progression of hepatocarcinogenesis and is associated with poorly differentiated histopathology and an invasive phenotype (7). Hedgehog signaling is triggered when Hh proteins (like Sonic, Indian, Desert) bind to the PTCH receptor, releasing the inhibition on Smoothened (Smo). Smo then activates Gli transcription factors, especially Gli1, which move to the nucleus to trigger the expression of target genes (8, 9). Hedgehog signaling is crucial in modulating drug resistance through ABC transporters, such as ABCC1 which contributing to drug resistance particularly SOR resistance in HCC patients (10–12).

Several studies revealed that natural products offer promising, multi-faceted approaches to overcome the challenge of drug resistance in therapy in cancer (13–16). Ashwagandha (*Withania somnifera L.*,* Solanaceae*) is a natural herb investigated for its potential in treating various conditions, including cancer (17). The primary active constituents of the plant that have been identified as bioactive are withanolides A-Y, withaferin A, withasomniferin A, withasomnidienone, withasomnier A–C, withanone, etc. In addition to lactones, the plant extract also contains alkaloids such as isopelletierine, anaferine, cuseohygrine, and anahygrine (18).

Previous research has shown that Ashwagandha extract is a potent antioxidant with the ability to inhibit cancer cell growth, suggesting its potential as an effective and economical anticancer agent (17). Recently, it was demonstrated that withanolide glycosides from ashwagandha trigger apoptosis in liver cancer cells through caspase activation and Bcl-2/Bax balance disruption, and inhibit angiogenesis by blocking vascular endothelial growth factor receptor2 signaling in endothelial cells (19). Therefore, this study hypothesizes that the whole ASH-AE may suppress hepatocellular carcinoma cell proliferation and resistance through modulation of Hedgehog signaling and CSC-related markers.

## Materials and methods

The present study was an in vitro study employing the human hepatocellular carcinoma cell line HepG2 obtained from ATCC (HB-8065 ™). The study was conducted at the Center of Excellence for Research in Regenerative Medicine and Applications (CERRMA) at Alexandria Faculty of Medicine. This study was approved by ethical committee of Medical Research Institute, Alexandria university (E/C. S/N. T1/2023). The obtained cells were divided into 4 groups according to the treatment regimen received. **Group I**: HepG2 cells cultured in drug free environment as untreated control. **Group II**: HepG2 cells are treated with different concentrations of SOR. **Group III**: HepG2 cells treated with different concentrations of ASH-AE. **Group IV**: HepG2 cells treated with different concentrations of SOR and ASH-AE.

### Drugs Preparation

#### Sorafenib

A stock solution of SOR (20 mM) was prepared by dissolving one capsule of SOR (Nexavar 200 mg) in 21.5 ml Dimethyl sulfoxide (DMSO). The solution was sterilized by passage through a 0.22 μm Millipore filter. The stock solution was aliquoted and diluted to give the desired working concentrations based on preliminary cytotoxicity testing (4.3–34.2 µM) using complete cell culture medium as diluent.

#### ASH-AE

##### Preparation of ASH-AE

The plant was collected from the Alexandria tramway in Egypt in accordance with environmental safeguards. The samples were deposited as voucher specimens in Alexandria University herbarium (ALEX) and identified according to Boulos, L. (2002) (20). The whole plant was air-dried for 48 h then, it was oven-dried at 60 °C for two days. After drying, the plant was ground and stored in an airtight glass container at room temperature. 5 gm of the powder was infused in 250 ml (1:50 w/v) of freshly boiled double distilled water for 25 min. After that, the infusion was left to cool to room temperature and centrifuged at 12,000 rpm for 15 min. The supernatants were recentrifuged at 12,000 rpm for 10 min (21). The supernatants were then dried by lyophilization (22). The yield of the dried aqueous extract (AE) was 1.4 g. Next, a stock solution of ASH-AE (100 mg/ml) was prepared, sterilized by passage through a 0.22 μm Millipore filter and diluted to give the desired working concentrations based on preliminary cytotoxicity testing (1.3–10.4 mg/ml) using complete cell culture medium as diluent.

##### Characterization of ASH-AE

Gas chromatography-mass spectrometry (GC-MS) and high-performance liquid chromatography (HPLC) were used for the identification of ASH-AE components.

###### GC-MS

The chemical composition of ASH-AE was identified using Trace GC1310-15Q mass spectrometer (Thermo Scientific, Austin, TX, USA) with a direct capillary column TG-SMS (30 m x 0.25 mm x 0.25 μm film thickness) (23). The column oven temperature was initially held at 50 °C and then increased by 5 °C/min to 230 °C held for 2 min. Then, the temperature was increased to the final temperature of 290 C by 30 °C/min and held for 2 min. The injector and MS transfer line temperatures were kept at 250, 260 °C respectively, Helium was used as a carrier gas at a constant flow rate of 1 ml/min. The solvent delay was 3 min and diluted samples of 1 ul were injected automatically using Auto sampler (AS1300) coupled with GC in the split mode. Electron impact mass spectra were collected at 70 eV ionization voltages over the range of m/z 40–1000 in full scan mode. The ion source temperature was set at 200 °C. The components were identified by comparison of their retention times and mass spectra with those of WILEY 09 and NIST 11 mass spectral database.

###### HPLC

HPLC analysis of ASH-AE were performed by HPLC-(Agilent 1100) is composed of a two LC pumps pump, a UV/Vis detector. C18 column (250 mm × 4.60 mm, 5 μm particle size). Phenolic acids were separated by employing a mobile phase of two solvents 0.1% methanol: phosphoric acid (50: 50 v/v, isocratic mode). The flow rate was adjusted to 1.0 ml/min; the detector was set at 280 nm with the mobile phase (24). Flavonoids were separated by employing mobile phase consisted of a binary mixture of methanol/water (50:50 v/v) adjusted to pH 2.8 with phosphoric acid, at isocratic flow rate of 1.0 ml min^– 1^ (25). Alkaloids were separated by employing a mobile phase of mixture of acetonitrile: Methanol: Ortho phosphoric acid (55:45:1). The flow rate was adjusted to 1.0 ml/min; the detector was set at 280 nm with the mobile phase (26). Glycosides were analyzed by employing a mobile phase of mixture of water (1% methanol): mixture of [methanol: ethanol: isopropanol] (55:45:1) in (50:50). The flow rate was adjusted to 1.0 ml/min; the detector was set at 220 nm with the mobile phase (27). Chromatograms were obtained and analyzed using the Agilent ChemStation.

### Cell culture

All cell culture assays adopted in the present work were performed under strictly aseptic conditions. Hepatocellular cancer-derived HepG2 cells were maintained throughout the study in high glucose Dulbecco`s modified Eagle`s Medium (DMEM) (Lonza, Belgium) with 2 mM L-glutamine supplemented with 10% (v/v) heat-inactivated fetal bovine serum (FBS, Sigma, USA), 100 IU/ml penicillin and 100 µg/ml streptomycin (Lonza, Belgium). All culture systems were basically carried out in a humidified CO_2_ incubator at 37 °C with a permanent atmosphere of 5% CO_2_ and 95% air.

### Assessment of cell viability

The viability of drug-treated and untreated HepG2 cells was determined by the 3- [4, 5 dimethylthiazol-2-yl]−2, 5-diphenyl tetrazolium bromide (MTT) cell proliferation and viability assay. Cells were seeded in sterile flat-bottomed 96-well plate at a density of 7 × 10^3^ cells/well and incubated for 24 h in a humidified 5% CO_2_ incubator at 37 °C. After 24 h cells were treated with SOR and ASH-AE, either alone or in combination for 48 h, at concentrations of 4.3, 8.7, 17.4, 26.1 and 34.2 µM for SOR, 1.3, 2.6, 5.2, 7.8 and 10.4 mg/ml for ASH-AE, all in triplicates. After 48 h, the culture media were aspirated, replaced by 100 µl of new media with (0.5 mg/ml) of MTT solution and incubated for 4 h. After incubation, the media was removed and 100 µl DMSO/well was added and gently rocked in the dark by an ELISA shaker for 20 min. Lastly, after the dissolution of formazan blue crystals in DMSO, the absorbance at 570 nm was measured by ELISA well-plate reader (Tecan, Infinite F50, M€annedorf, Switzerland). The percentages of cell viability were calculated as the ratio of treated to untreated cell absorbance (28). The results were analyzed using CompuSyn for determination of drug concentrations equivalent to 50% inhibition of cell proliferation in drug-treated cells compared to untreated control (IC_50_) for SOR and/or ASH-AE and also for determining synergistic, antagonistic or additive effects of the two drugs by calculating the combination index (CI) where, CI > 1 indicates antagonism, CI = 1 indicates an additive effect and CI < 1 indicates synergism (29).

### Flow cytometry technique

The protein expression of CD90 in HepG2 cells was determined by flow cytometry technique. HepG2 cells were seeded in 6-well tissue culture plates at density of 3 × 10^5^ cells/well and incubated in a humidified 5% CO_2_ incubator at 37 °C. After 24 h, cells were treated with IC50 of SOR and/or ASH-AE in triplicates and incubated in the same conditions for 48 h. After that, cells were collected and 1 × 10^6^ cells were resuspended in 100 µl PBS and then incubated with PE/Elab Fluor^®^ 594 Anti-Human CD90 Antibody (1:50 dilution) (Elabscience, USA) for 30 min at room temperature. After washing cells by 2 ml PBS, cells were resuspended by 500 µl PBS buffer and analyzed by using BD FACS Calibur flow cytometer and cell sorter and Cell Quest™ software.

### Gene expression analysis

HepG2 cells were seeded in 6-well tissue culture plates at density of 3 × 10^5^ cells/well in complete tissue culture medium and incubated in a humidified 5% CO2 incubator at 37 °C. After 24 h, cells were treated with IC50 of SOR and/or ASH-AE in triplicates and incubated in the same conditions for 48 h. Then, total RNA was extracted from harvested HepG2 cells using RNeasy Mini Kit (Qiagen, Germany). The concentration of extracted RNA was measured by measuring the absorbance at 260 nm (A260) using a NanoDrop 2000 Spectrophotometer (Thermo Scientific, USA). The ratio of absorbance values at 260 nm and 280 nm (A260/A280) was used to determine RNA purity. The A260/A280 ratio of pure RNA is 1.9–2.1. The expression of SHh, PTCH1 and ABCC1 genes was determined by quantitative reverse transcription polymerase chain reaction (qRT-PCR) using TOPreal One-step RT qPCR Kit (Enzynomics, Korea) according to manufacturer’s protocols and Real-time PCR thermal cycler (Qiagen Rotor-Gene Q system). The forward and reverse primers for each target gene were shown in Table 1 and the primer assay GAPDH which was served as reference gene was ready made (QuantiTect Primer Assay, Qiagen, Germany). Results were analyzed using the comparative cycle threshold (ΔΔCt) method for calculating fold change of gene expression. 

**Table 1:** Primer assays for target genes

Gene	Primer sequence
SHh	Forward: 5'CCCGACATCATATTTAAGGATGAAGA3' Reverse: 5'AAGCGTTCAACTTGTCCTTAC3'
PTCH1	Forward: 5'AAACAGGTTACATGGATCAGATAATAG3' Reverse: 5'CCCTTCCCAGAAGCAGT3'
ABCC1	Forward: 5'ACCCTAATCCCTGCCCAGAG-3' Reverse: 5'CGCATTCCTTCTTCCAGTTC-3'

### ICC technique

The protein expression and localization of Gli1 in HepG2 cells were determined by ICC. HepG2 cells were seeded on coverslips in 6-well tissue culture plates at density of 2 × 10^5^ cells/well and incubated in a humidified 5% CO2 incubator at 37 °C. After 24 h, cells were treated with IC50 of SOR and/or ASH-AE in triplicates and incubated in the same conditions for 48 h. Then, the cells were fixed with 4% neutral formalin at room temperature then washed three times with PBS each for 5 mins. Permeabilization of cells was done using 0.2% triton x for 15 min then washed three times with PBS each for 5 mins. The nonspecific antibodies were blocked by adding 0.2% BSA and incubated for 1 h then washed three times with PBS each for 5 mins. After that rabbit polyclonal anti-Gli1 antibody (1:500 dilution with 1% BSA) (MyBioscience, USA) was added and incubated overnight then washed three times with PBS each for 5 mins. Goat Anti-Rabbit IgG H&L (Alexa Flour 488, ab150077) secondary antibody was then added and incubated for 1 h in dark then washed three times with PBS each for 5 mins. Cover slips were then mounted by water and fixed on slides and examined using Confocal Laser Scanning Microscopy. The captured images were analyzed by image j software and the percentage of nuclear translocation of Gli1 in all studied groups was calculated using the following equation:

$$\:\mathrm{\%}\:\mathrm{N}\mathrm{u}\mathrm{c}\mathrm{l}\mathrm{e}\mathrm{a}\mathrm{r}\:\mathrm{t}\mathrm{r}\mathrm{a}\mathrm{n}\mathrm{s}\mathrm{l}\mathrm{o}\mathrm{c}\mathrm{a}\mathrm{t}\mathrm{i}\mathrm{o}\mathrm{n}\:\mathrm{o}\mathrm{f}\:\mathrm{G}\mathrm{l}\mathrm{i}1=\:\frac{\mathrm{N}\mathrm{u}\mathrm{m}\mathrm{b}\mathrm{e}\mathrm{r}\:\mathrm{o}\mathrm{f}\:\mathrm{f}\mathrm{l}\mathrm{u}\mathrm{o}\mathrm{r}\mathrm{e}\mathrm{s}\mathrm{c}\mathrm{e}\mathrm{n}\mathrm{t}\:\mathrm{p}\mathrm{a}\mathrm{r}\mathrm{t}\mathrm{i}\mathrm{c}\mathrm{l}\mathrm{e}\mathrm{s}\:\mathrm{i}\mathrm{n}\:\mathrm{n}\mathrm{u}\mathrm{c}\mathrm{l}\mathrm{e}\mathrm{i}}{\mathrm{T}\mathrm{o}\mathrm{t}\mathrm{a}\mathrm{l}\:\mathrm{n}\mathrm{u}\mathrm{m}\mathrm{b}\mathrm{e}\mathrm{r}\:\mathrm{o}\mathrm{f}\:\mathrm{f}\mathrm{l}\mathrm{u}\mathrm{o}\mathrm{r}\mathrm{e}\mathrm{s}\mathrm{c}\mathrm{e}\mathrm{n}\mathrm{t}\:\mathrm{p}\mathrm{a}\mathrm{r}\mathrm{t}\mathrm{i}\mathrm{c}\mathrm{l}\mathrm{e}\mathrm{s}}\times\:100$$

### Statistical analysis

Data were fed to the computer and analyzed using IBM SPSS software package version 20.0. The distributions of quantitative variables were tested for normality using the Shapiro-Wilk test. Since the variables reveal normal distribution, a parametric test was applied. Quantitative data were described by mean and standard deviation (SD). Comparison between studied groups was done using one-way analysis of variance (ANOVA) test and multiple comparisons were done by post-hoc tests (Tukey and Games-howell). Significance of the results obtained was judged at the 5% level.

## Results

### GC-MS analysis of ASH-AE

**Fig. 1:**
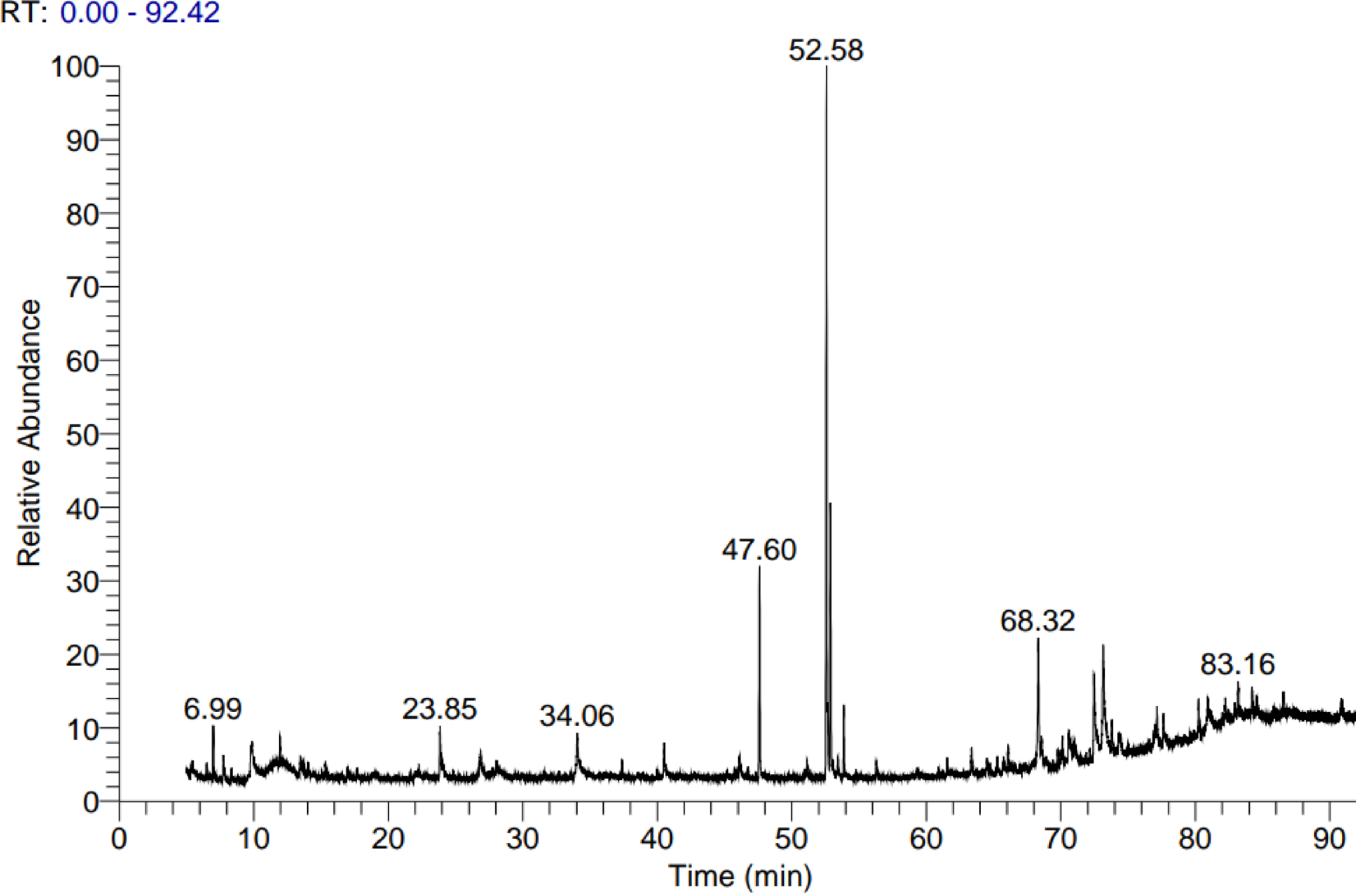
GC-MS analysis of bioactive compounds in ASH-AE

The total chromatogram of aqueous extract of the vegetative leaf, root, and stem of *Withania somnifera* was shown in Fig. 1, which represents retention times of all the components detected in the extract. The chemical composition of components of aqueous extract of the *W. somnifera* whole plant was indicated in Table 2.

**Table 2:** GC–MS analysis of bioactive compounds in ashwagandha aqueous extract

No	Nomenclature	Molecular formula	Chemical structure	RT	Area %
1	Lansoprazole	C16H14F3N3O2S		5.40	0.69
2	Trisiloxane, 1,1,3,3,5,5-Hexamethyl	C6H20O2Si3		9.87	1.01
3	Benzothieno[2,3-C] Quino Lin-6(5h)-One, 2-Methoxy	C16H11NO2S		11. 95	0.62
4	4h-1-Benzothiopyran-4- One, 3-[(4-Methylphenyl)Amin O]-, 1-Oxide	C16H13NO2S		16. 97	0.37
5	Cyclohexasiloxane, Dodecamethyl	C12H36O6Si6		26. 86	1.21
6	Cycloheptasiloxane, Tetradecamethyl	C14H42O7Si7		34. 05	2
7	Cyclooctasiloxane, Hexadecamethyl	C16H48O8Si8		40. 51	1.21
8	Hexadecanoic Acid, Methyl Ester	C17H34O2		47. 60	5.52
9	Octasiloxane, 1,1,3,3,5,5,7,7,9,9,11,11,13,13,1 5,15-Hexadecamethyl	C16H50O7Si8		50. 97	0.29
10	Cyclodecasiloxane, Eicosamethyl	C20H60O10Si10		51. 13	0.57
11	2,2,4,4,6,6,8,8,10,10,12,12,14,1 4,16,16,18,18,20,20-Icosame Thylcyclodecasiloxan E #	C20H60O10Si10		51. 13	0.57
12	9,12-Octadecadienoic Acid (Z,Z)-, 2,3-Bis[(Trimethylsilyl) Oxy]Propyl Ester	C27H54O4Si2		64. 76	0.24
13	9,12,15-Octadecatrienoic Acid, 2,3-Bis[(Trimethylsilyl) Oxy] Propyl Ester, (Z,Z,Z)	C27H52O4Si2		65. 28	0.63
14	5,11,17 23-Tetrakis (1,1-Dimethyl Ethyl)−28-Methoxypenta Cyclo[19.3.1.1(3,7)0.1(9,13)0.1 (15,19)]Octacosa-1(25),3,5, 7(28),9,11,13(27),15,17,19(26),2 1,23-Dodecene-25,26,27-Tri Ol	C45H58O4		68. 55	4.96
15	9,12-Octadecadienoic Acid (Z,Z)-, 2,3-Bis[(Trimethylsilyl) Oxy]Propyl Ester	C27H54O4Si2		69. 76	0.54
16	13-Docosenamide, (Z)	C22H43NO		72. 47	3.42
17	9,12-Octadecadienoic Acid (Z,Z)-, 2,3-Bis[(Trimethylsilyl) Oxy]Propyl Ester	C27H54O4Si2		74. 33	0.75
18	4h-1-Benzopyran-4-One, 2-(3,4-Dihydroxyphenyl) −6,8-Di-Á-D-Glucopyrano Syl-5,7-Dihydroxy	C27H30O16		75. 00	0.68
19	Octasiloxane, 1,1,3,3,5,5,7,7,9,9,11,11,13,13,1 5,15-Hexadecamethyl	C16H50O7Si8		80. 93	0.37

### HPLC analysis of ASH-AE

The fractionation of the phenolic compounds, flavonoids and alkaloids in ASH-AE extract by HPLC revealed that ASH-AE contains 7 phenolic compounds, 7 flavonoid compounds and 3 alkaloids compounds were shown in Table 3. The major phenolic compounds are P- coumaric (15.68 µg/gm), caffeic acid (14.52 µg/gm), ferulic (13.95 µg/gm) and catechol (8.31 µg/gm). The major flavonoids are rutin (17.49 µg/gm), Luteolin (17.36 µg/gm) and quercetin (7.42 µg/gm). The Alkaloids in ASH-AE are withanolide (22.1 µg/gm), withaferin-A (15.56 µg/gm) and withanone (10.68 µg/gm).

**Table 3:** HPLC analysis of ASH-AE

Class	Pack name	Concentration(µg/gm)	Rt
Phenolic compounds	P- Coumaric	15.68	6.0
	Caffeic	14.52	8.0
	Ferulic	13.95	11.0
	Catechol	8.31	4.0
	Syringic	7.82	5.0
	Gallic	7.65	10.0
	Cinnamic	3.20	7.0
Flavonoids compounds	Rutin	18.50	5.0
	Luteolin	17.36	9.0
	Quercetin	7.42	7.0
	Naringin	6.36	4.6
	Catechin	4.90	12.0
	Kaempferol	3.74	8.0
	Apigenin	3.10	10.0
Alkaloids	withaferin-A	15.56	3.5
	Withanone	10.68	8.0
	Withanolides	22.1	8.0

### The effect of SOR and/or ASH-AE on viability of HepG2 cells

**Fig. 2:**
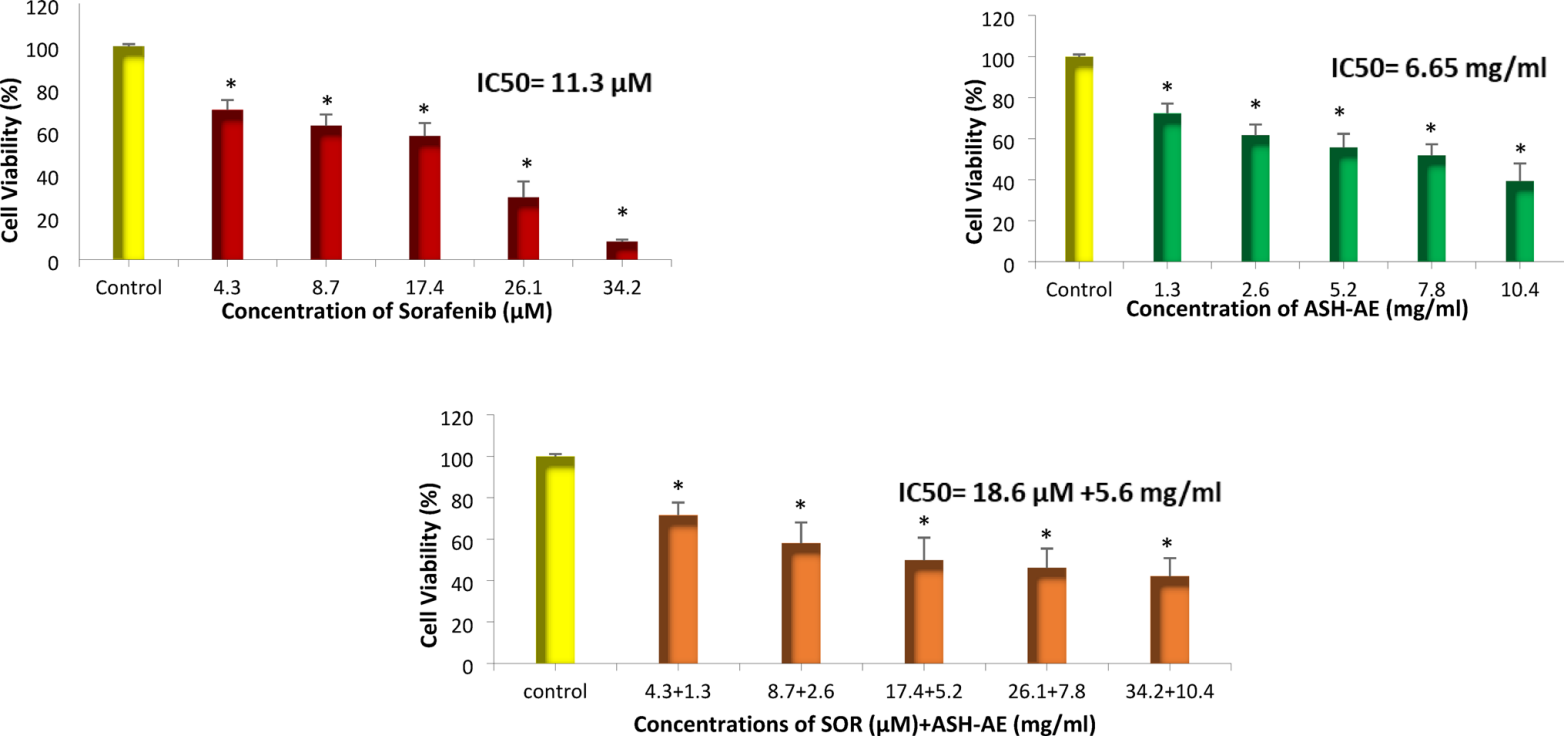
Bar chart representing the viability (%) of HepG2 cells treated with different concentrations of sorafenib and/or ashwagandha aqueous extract for 48 h. *: Statistically significant difference from control group at *p* ≤ 0.05

**Fig. 3:**
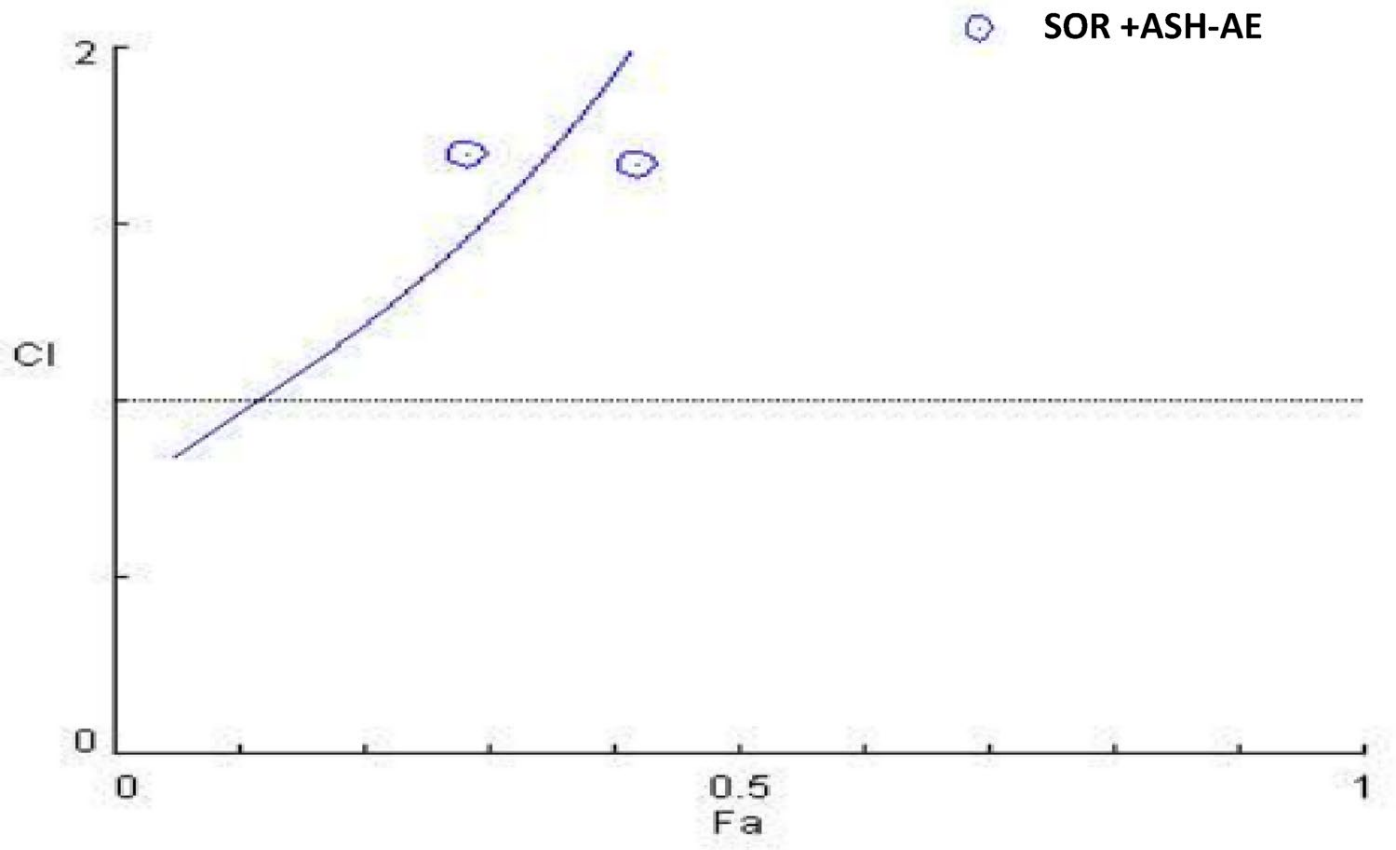
Combination index plot for SOR and ASH-AE in HepG2 cells

Mean values of viability (%) was 100 for untreated cells (control); 70.23, 62.79, 58.03, 29.15, 8.53 for HepG2 cells treated with different concentrations of SOR (4.3, 8.7, 17.4, 26.1 and 34. 2 µM, respectively); 72.29, 61.58, 55.74, 51.75, 39.31 for cells treated with different concentrations of ASH-AE (1.3, 2.6, 5.2, 7.8 and 10.4 mg/ml, respectively) and 71.57, 58.04, 49.84, 46.06, 42.19 for cells treated with different concentrations of SOR and ASH-AE in combination (4.3 + 1.3, 8.7 + 2.6, 17.4 + 5.2, 26.1 + 7.8 and 34.2 µM + 10.4 mg/ml, respectively). The IC_50_ values of SOR and/or ASH-AE were 11.3 µM, 6.65 mg/ml and 18.6 µM + 5.6 mg/ml, respectively. The statistical analysis of these results revealed that HepG2 cell viability (%) in groups treated with SOR and/or ASH-AE was significantly lower than that in untreated groups in a concentration dependent manner (P ˂0.001) (Fig. 2). CI was 2.5 indicating that combination of SOR and ASH-AE generates antagonistic antitumor effect in HepG2 cells (Fig. 3).

### The effect of SOR and/or ASH-AE on CD90 protein levels

**Fig. 4:**
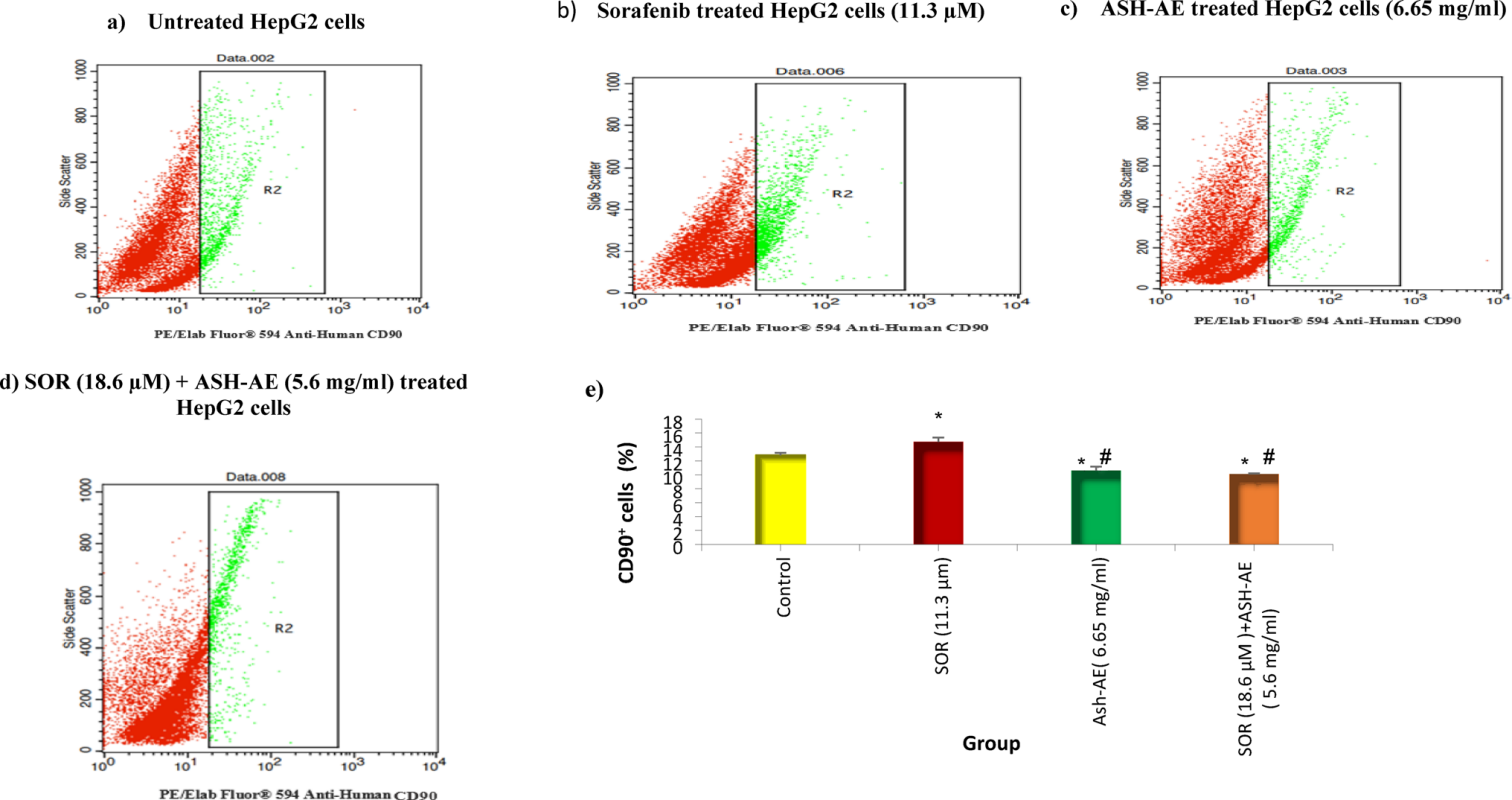
Flow cytometry analysis of CD90 expression in HepG2 cells treated with SOR and/or ASH-AE for 48 h. *: Statistically significant difference from control group at *p* ≤ 0.05, #: Statistically significant difference from SOR group at *p* ≤ 0.05

Mean values of CD90^+^ cells (%) was 12.79 in untreated HepG2 cells; 14.73 in cells treated with 11.3 µM SOR; 10.6 in cells treated with 6.65 mg/ml ASH-AE; and 10.1 in cells treated with combination of 18.6 µM SOR and 5.6 mg/ml ASH-AE. The statistical analysis of these results indicated that the percentage of CD90^+^ cells in cells treated with SOR was significantly higher than that in untreated cells (*P* = 0.005), while it was significantly lower than that in untreated cells in cells treated with ASH-AE alone or in combination with SOR (*P* = 0.002 and P 0.001, respectively). Moreover, the percentage of CD90^+^ cells treated with SOR was significantly higher than that in cells treated with ASH-AE alone or in combination with SOR (*P* < 0.001). On the other hand, there was no significant difference between percentage of CD90^+^ cells treated with ASH-AE and that treated with combination of SOR and ASH-AE (*P* = 0.597) (Fig. 4).

### The effect of SOR and/or ASH-AE on gene expression of SHh, PTCH and ABCC1

The mean fold change of SHh, PTCH1 and ABCC1 in all studied groups were shown in Table 4 and illustrated in Fig. 5. The statistical analysis of these results revealed that gene expression of SHh and PTCH1 in cells treated with SOR was insignificantly lower than that in untreated cells (*P* = 0.4 and 0.5, respectively), while it was significantly lower than that in untreated cells in cells treated with ASH-AE alone or in combination with SOR (*P* = 0.001 and 0.005, respectively). Moreover, gene expression of SHh and PTCH1in cells treated with SOR was significantly higher than that in cells treated with ASH-AE alone or in combination with SOR (*P* = 0.035 and 0.03, respectively). On the other hand, there was insignificant difference between SHh and PTCH1gene expressions in cells treated with ASH-AE alone and that treated with combination of SOR and ASH-AE (*P* = 0.999 and 0.3, respectively). Regarding ABCC1, gene expression of ABCC1 in cells treated with SOR was significantly higher than that in untreated cells (*P* = 0.004), while it was significantly lower than that in untreated cells in cells treated with ASH-AE alone or in combination with SOR (*P* = 0.02 and 0.01, respectively). Moreover, gene expression of ABCC1 in cells treated with SOR was significantly higher than that in cells treated with ASH-AE alone or in combination with SOR (*P* = 0.006 and 0.002, respectively). On the other hand, there was insignificant difference between ABCC1 gene expression in cells treated with ASH-AE alone and that treated with combination of SOR and ASH-AE (*P* = 0.7).

**Table 4:** Statistical analysis of SHh, PATCH1 and ABCC1 gene expression in all studied groups

	Control	SOR (11.3 µM)	ASH-AE (6.65 mg/ml)	SOR + ASH-AE (18.6 µM + 5.6 mg/ml)	F	P
SHh gene						
Mean ± SD	1 ± 0.04	0.8 ± 0.15	0.1 ± 0.01	0.1 ± 0.01	262.62	˂0.001*
P1		0.4	0.001*	0.001*		
P2			0.035^#^	0.035^#^		
P3				0.999		
PATCH1 gene						
Mean ± SD	1 ± 0.07	0.8 ± 0.15	0.02 ± 0.004	0.03 ± 0.01	119.384	˂0.001*
P1		0.5	0.005*	0.005*		
P2			0.03^#^	0.03^#^		
P3				0.3		
ABCC1 gene						
Mean ± SD	1.09 ± 0.16	2.2 ± 0.17	0.19 ± 0.01	0.2 ± 0.07	132.53	˂0.001*
P1		0.004*	0.02*	0.01*		
P2			0.006^#^	0.002^#^		
P3				0.7		

F: F for ANOVA test, pairwise comparisons were done using post hoc test (Games-Howell).

P: P value for comparing between all studied groups.

P1: P value for comparing between control group and each other group.

P2: P value for comparing between SOR group and groups treated with ASH-AE alone and in combination with SOR.

P3: P value for comparing between groups treated with ASH-AE alone and in combination with SOR.

*: Statistically significant difference from control group at p ≤ 0.05.

#: Statistically significant difference from SOR group at p ≤ 0.05.

**Fig. 5:**
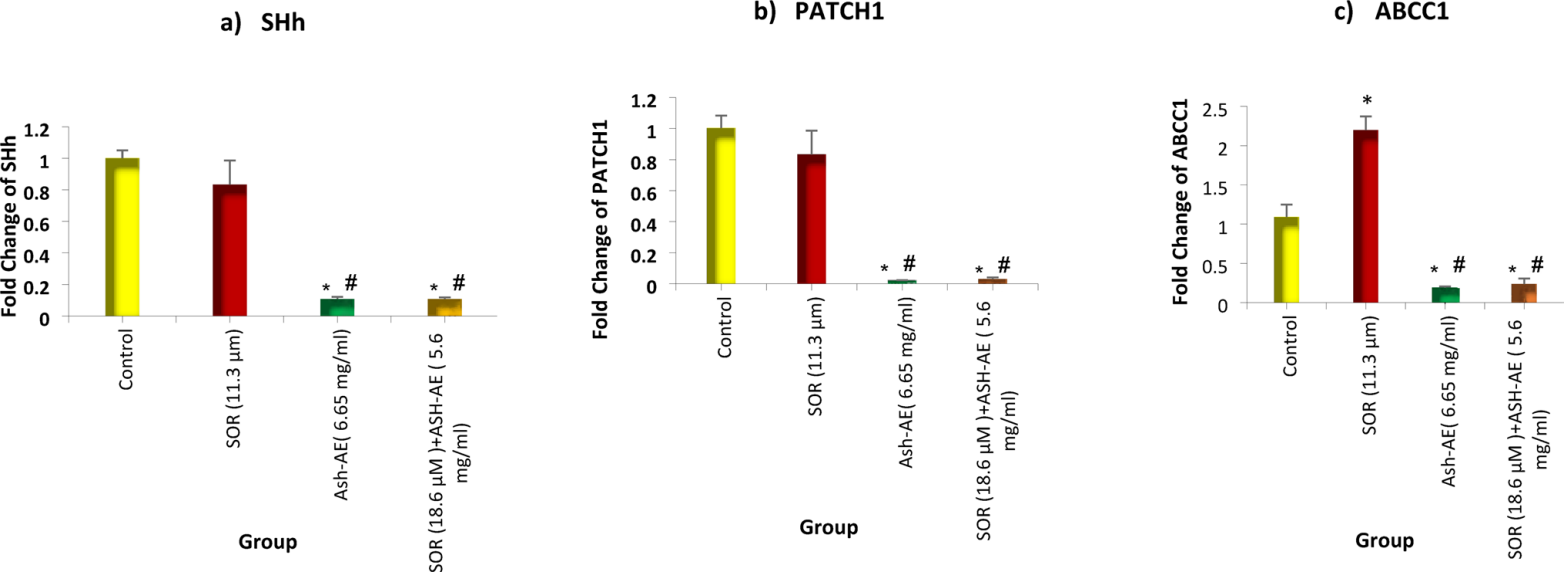
Bar chart representing the effect of SOR and/or ASH-AE on gene expression of (**a**) SHh, (**b**) PATCH1and (**c**) ABCC1 in HepG2 cells. *: Statistically significant difference from control group at *p* ≤ 0.05, #: Statistically significant difference from SOR group at *p* ≤ 0.05

### The effect of SOR and/or ASH-AE on the Gli1 protein expression and nuclear translocation

**Fig. 6:**
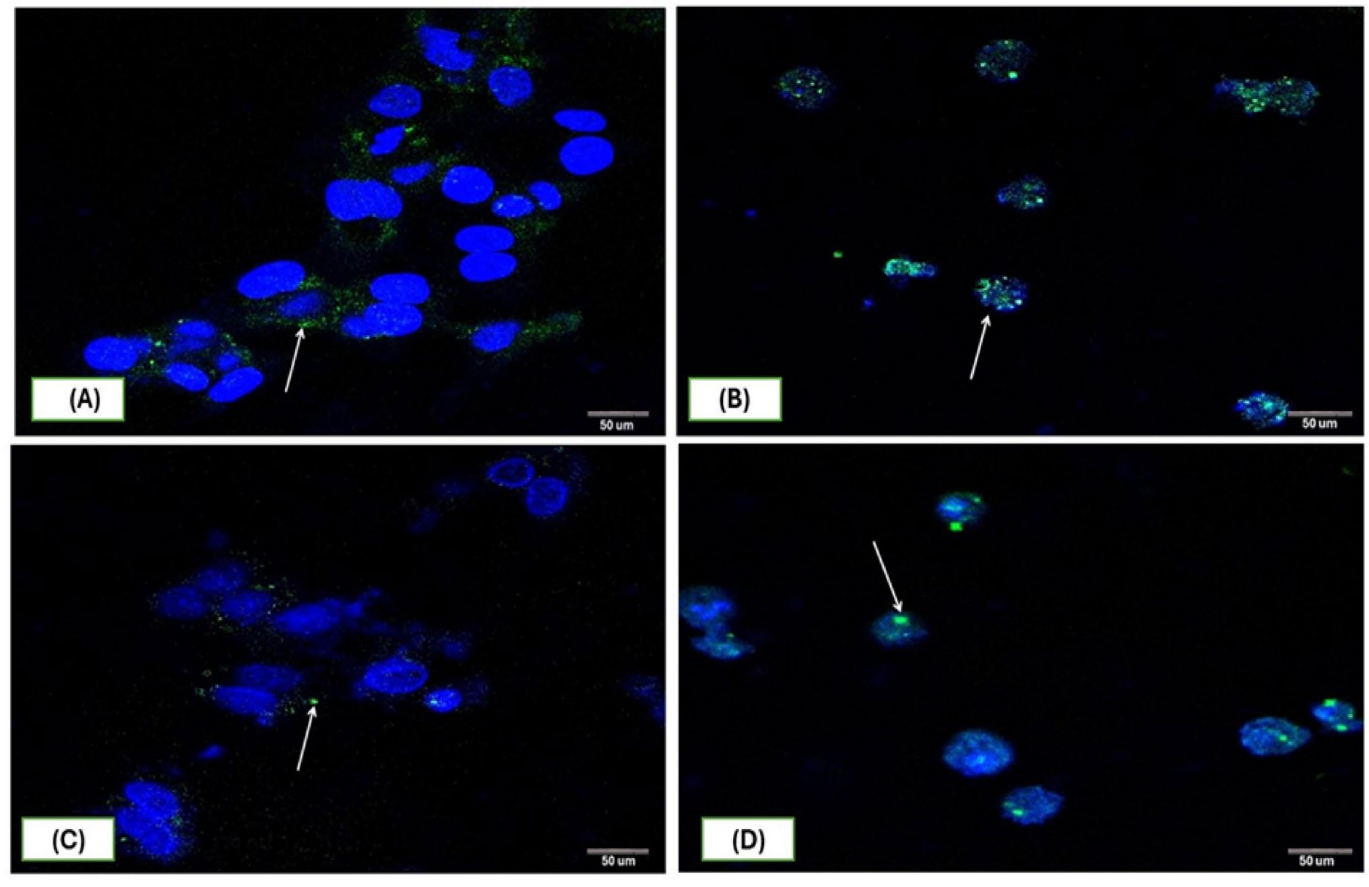
Representative confocal immunofluorescence images of Hepg2 cells with immunoreactivity of Gli1 showing different positivity as following: (**A**) Strong positive (↑) immune reaction in untreated HepG2 cells, (**B**) Strong positive immune reaction in HepG2 cells treated with SOR. Note: translocated Gli1 to the nucleus (↑), (**C**) Weak positive (↑) immune reaction in HepG2 cells treated with ASH-AE, (**D**) Moderate positive (↑) immune reaction in HepG2 cells treated with SOR + ASH-AE

Immunofluorescence detection of Gli1 examined by Confocal Laser Scanning Microscope displayed strong immunopositivity in untreated HepG2 cells group (Fig. 6A) and strong immunopositivity in cells treated with SOR with many Gli1 nuclear translocation (Fig. 6B). Weak immunopositivity was observed after 48 h. in ASH-AE group (Fig. 6C). HepG2 cells treated with SOR in combination with ASH-AE showed moderate positive immune reaction (Fig. 6D).

**Fig. 7:**
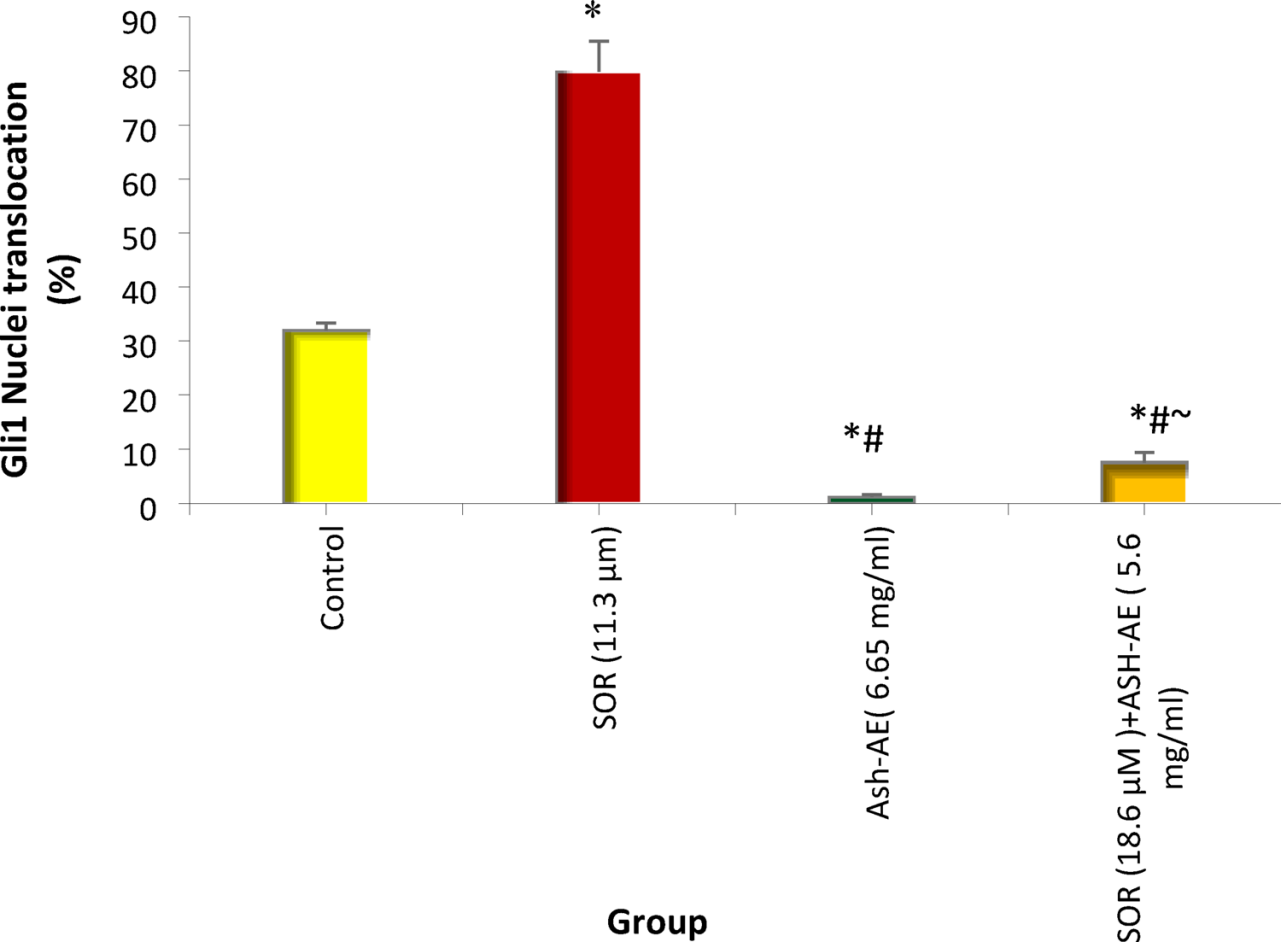
Bar chart representing the effect of SOR and/or ASH-AE on the percentage of Gli1 nuclear translocation in HepG2 cells. *: Statistically significant difference from control group at *p* ≤ 0.05, #: Statistically significant difference from SOR group at *p* ≤ 0.05, ⁓: Statistically significant difference from ASH-AE group at *p* ≤ 0.05

Mean values of Gli1 nuclear translocation (%) was 31.9 in untreated HepG2 cells; 79.9 in cells treated with 11.3 µM SOR; 1.21 in cells treated with 6.65 mg/ml ASH-AE; and 7.45 in cells treated with combination of 18.6 µM SOR and 5.6 mg/ml ASH-AE. The statistical analysis of these results indicated that the percentage of Gli1 nuclear translocation in cells treated with SOR was significantly higher than that in untreated cells (*P* < 0.001), while it was significantly lower than that in untreated cells in cells treated with ASH-AE either alone or in combination with SOR (P ˂0.001). Moreover, the percentage of Gli1 nuclear translocation in cells treated with SOR was significantly higher than that in cells treated with ASH-AE alone or in combination with SOR (*P* < 0.001). Additionally, there was significant difference between the percentage of Gli1 nuclear translocation in cells treated with ASH-AE and that treated with combination of SOR and ASH-AE (*P* = 0.005) (Fig. 7).

## Discussion

Ashwagandha (*Withania somnifera*) has gained widespread recognition for its broad range of health benefits, including its potential cancer-preventive and therapeutic effects. Despite substantial research supporting the efficacy of this herb, its clinical application in cancer treatment remains limited. This limitation primarily stems from the inconsistent therapeutic outcomes associated with the variable composition and concentration of active compounds in the plant extract, which affects the herb’s pharmacological properties (30). Results of phytochemical investigation using Gc-MS designated 19 compounds as the dominant compounds in vegetative leaf, root, and stem of *Withania somnifera.* The major compounds based on their concentration and antibacterial as well as anticancer potentials were Cyclooctasiloxane hexadecamethyl, Hexadecanoic Acid, Methyl Ester, 9,12-Octadecadienoic acid (z, z)-, 2,3-bis[(trimethylsilyl)oxy]propyl ester and Cycloheptasiloxane, tetradecamethyl (31–34). The HPLC analysis revealed high concentrations of withanoloides and withaferin A in the aqueous extract of the stem, leaf, and root of *Withania somnifera*, these compounds known for their anti-cancer, neuroprotective, and anti-stress properties. These bioactive constituents are also associated with memory recovery, anti-mutagenic effects, and inhibition of cancer cell proliferation, including lungs, colon, and breast cancer cells. Additionally, they act as growth inhibitors in human tumor cell lines, counteracting mutagenic effects, and enhancing the cellular immune response to mitogens. Furthermore, they have been shown to reverse paclitaxel-induced neutropenia, are recognized as potent radiosensitizers in chemotherapy, and demonstrate efficacy in combating melanoma-induced metastasis (35). Also, ASH-AE contains 7 phenolic compounds, 7 flavonoid compounds and one other alkaloid (Withanone) compound which showed good cytotoxic properties against HepG2 cells. This finding is consistent with Moustafa et al., study which showed that withaferin A and withanolide are the major active components in ASH-AE (36).

The results of the current study indicated that ASH-AE alone or in combination with SOR significantly reduced HepG2 cell viability in a concentration dependent manner. This finding is consistent with previous studies indicated that ASH-AE either from roots or leaves decreased viability of many types of cancer cell lines including breast cancer cell lines and HepG2 cells mainly through apoptosis induction and its antioxidant effect (17, 37, 38). Additionally, withaferin-A, which is one of the main active components of ASH-AE, causes G 2/M cell cycle arrest, as well as modulation of cyclin B1, p34 (cell division cycle 2 protein), proliferating cell nuclear antigen (PCNA) levels, and signal transducer and activator of transcription 3 (STAT3) levels. In addition, its phosphorylation and influence on p53 levels induce the apoptotic markers Bcl-2-associated X protein (BAX), and caspase-3 (39). Recently, it was demonstrated that withanolide glycosides exhibit apoptotic and anti-angiogenic effect in HCC cells (19). Thus, the cytotoxic effect of ASH-AE against HepG2 cells may be attributed to its bioactive components including withaferin-A and withanolide. This finding suggests that ASH-AE may be an effective therapeutic agent against HCC. On the other hand, the results of this study revealed that the combination of SOR and ASH-AE generates antagonistic antitumor effect in HepG2 cells, this indicates that ASH-AE can’t be used with SOR, but it may be used with other therapeutic agents in treatment of HCC.

Liver CSCs, represented by CD44, CD133, Epithelial cell adhesion molecule or CD90-positive cells, have been proposed to be a key regulator of sorafenib resistance in HCC (40). The results of the present study indicated that SOR significantly increased the percentage of CD90^+^ cells compared to untreated cells. This finding supports the results of Cakil1 et al., who indicated that sorafenib increased CD90 protein expression in HepG2 cells (41). It was reported that high doses of sorafenib can cause acquired drug resistance and side effects. The low dose sorafenib treatment was associated with significant tumor growth delay in comparison to placebo and high-dose sorafenib groups in HCC, potentially reinforcing a preference for long-term low-dose treatment (42). On the other hand, ASH-AE alone or in combination with SOR significantly decreased the percentage of CD90^+^ cells compared to the untreated cells and that treated with SOR. Previous studies indicated that withaferin A significantly reduced stemness markers, and promoted the expression of differentiation markers in ovarian (43), gastrointestinal (44) and multiple myeloma cancer stem cells (45). Recently, it was reported that withaferin A decreased the expression of CSCs markers including CD90 in HepG2 cells and this effect was mediated by inhibiting the Phosphoinositide 3-kinase/Protein Kinase B (PI3K/AKT) pathway through microRNA-200c (46). This finding suggests that ASH-AE can target liver CSCs through downregulating CD90 expression by the action of withaferin A.

Activation of Hh signaling has been demonstrated to be a key factor in the development and progression of many cancers, including skin (47), gastrointestinal cancers (48) and HCC (49). Also, the aberrant activation of the hedgehog signaling pathway contributes to the maintenance of stemness in CSCs and the acquisition of EMT (50). Additionally, it was found that Gli1 mRNA overexpression in HCC tissues was associated with rapid recurrence of HCC tumors after surgery (49). Therefore, targeting hedgehog pathway can reduce CSCs population and overcome drug resistance. The results of the present study indicated that SOR significantly increased the expression and nuclear translocation of Gli in HepG2 cells compared to untreated cells. This is in agreement with Wang et al. who showed that sorafenib promotes the activation of hedgehog signaling pathway in HCC cells. Also, they demonstrated that sorafenib downregulated Mitogen-activated protein/extracellular signal-regulated kinase kinase kinase 2/3 (MEKK2/3) in HepG2 cells (51). MEKK2/3 was found to enhance the suppressor of fused sufu-Gli1 interaction, leading to Gli1 retention in cytoplasm, which occurs mechanistically via Gli1 phosphorylation by MEKK2/3 at multiple serine/threonine (Ser/Thr) sites (52). These suggest that sorafenib may suppress MEKK2/3 to promote Hh signaling activation and nuclear translocation of Gli, resulting in the upregulation of stemness related genes and sorafenib resistance in HepG2 cells.

In contrast, ASH-AE alone or in combination with SOR significantly downregulated SHh and PTCH1 gene expression in HepG2 cells compared to untreated cells and that treated with SOR. Also, it significantly decreased the expression and nuclear translocation of Gli1 in HepG2 cells compared to untreated cells and that treated with SOR. These findings are in accordance with previous study revealed significant decrease in SHh, PTCH-1, and Gli1 expression in Lung cancer tissues of ASH extract treated rats compared to lung cancer tissues of untreated rats (36). Moreover, it was reported that withanolide, one of the major active components of ASH aqueous extract resulted in inhibition of hedgehog signaling pathway in many types of cancer cells including pancreatic, prostate, and breast cancer cells (53). Significant positive correlation was reported between Hh signal and TGF-β1 in breast cancer, where high levels of TGF-β1 significantly increased the expression of SHh/Gli1 axis and EMT markers and revokes cell malignancy including migration and invasion (54, 55) Additionally, it was reported that treatment with ASH-extract was significantly decreased the expression of TGF-β1 as well as inhibited hedgehog signaling pathway in lung cancer tissues in vivo (36). Collectively, these suggest that ASH-AE which contain high concentration of withanolide can target hedgehog signaling pathway through downregulation of TGF-β1. On the other hand, nuclear translocation of Gli1 was significantly increased in cells treated with combination of SOR and ASH-AE compared to that treated with ASH-AE alone, this may be due to the strong activating effect of SOR on Gli in HepG2 cells.

Classically, ABCC1 overexpression is associated with tumor resistance to multiple chemotherapeutic agents. In HCC, the upregulation of ABCC1 expression could lead to tumor resistance to sorafenib and enhance the capacity for metastasis (56). The results of the current study indicated that SOR significantly upregulated ABCC1 expression in HepG2 cells compared to untreated cells. It was reported that ubiquitin specific peptidase 22 (USB22) promotes ABCC1 expression in HCC cells by activating the sirtuin 1/protein kinase B/multidrug resistance-associated protein 1 (SIRT1/Akt/MRP1) pathway (57). Also, previous study showed that USP22 upregulated ABCC1 expression and it was associated with sorafenib resistance in HCC cells (58). Moreover, previous studies reported that activation of hedgehog pathway in HCC cells resulting in therapy resistance through enhancing, invasive capability, EMT acquisition, and increasing the expression of ABC transporters including ABCC1 (10) and ABCB2 (59). These suggest that upregulation of ABCC1 in SOR-treated cells may be due to upregulation of USP22 expression or by activation of hedgehog signaling pathway as observed in the current study. On the other hand, ASH-AE alone or in combination with SOR significantly downregulated ABCC1 expression in HepG2 cells compared to untreated cells. This finding is similar to Moustafa et al., study which revealed that ASH extract decreased ABCG2 expression in lung cancer tissues of ASH extract treated rats compared to lung cancer tissues of untreated rats (36). This can be explained by downregulation of hedgehog signaling pathways in cells treated with ASH-AE alone or in combination with SOR as mentioned previously.

Collectively, the results of this study revealed that sorafenib increases CSCs markers while, ASH-AE decreases them, this highlights a potential conflict in their mechanisms of action, which can explain the antagonism. The two agents may act on different, but related, pathways that ultimately counteract each other when combined. For example, Ashwagandha’s mechanism to decrease CSC markers might interfere with a process necessary for sorafenib to exert its full toxic effect. Thus, further studies should be conducted to investigate the mechanism of antagonistic anticancer action of sorafenib and ASH-AE.

## Conclusion

From this study we concluded for the first time that Ashwagandha aqueous extract can inhibit proliferation of HepG2 cells and mitigate sorafenib-induced resistance-associated markers by targeting CD90^+^ cells through downregulating hedgehog signaling pathway, which is involved in tumor progression and drug resistance, suggesting that ASH-AE may be a promising therapeutic agent for hepatocellular carcinoma.

## Data Availability

The datasets used and/or analyzed during the current study are available from the corresponding author on reasonable request.

## Abbreviations

HCC: Hepatocellular carcinoma
CSCs: Cancer stem cells
CD90: Cluster of differentiation 90
SHh: Sonic Hedgehog
PTCH1: patched1
Gli1: glioma-associated oncogene
Smo: Smoothened
ABCC1: ATP-binding cassette subfamily C1
ASH-AE: Ashwagandha aqueous extract
SOR: Sorafenib
